# Comparison of Severe Acute Respiratory Syndrome Coronavirus 2 Screening Using Reverse Transcriptase–Quantitative Polymerase Chain Reaction or CRISPR-Based Assays in Asymptomatic College Students

**DOI:** 10.1001/jamanetworkopen.2020.37129

**Published:** 2021-02-11

**Authors:** Jennifer N. Rauch, Eric Valois, Jose Carlos Ponce-Rojas, Zach Aralis, Ryan S. Lach, Francesca Zappa, Morgane Audouard, Sabrina C. Solley, Chinmay Vaidya, Michael Costello, Holly Smith, Ali Javanbakht, Betsy Malear, Laura Polito, Stewart Comer, Katherine Arn, Kenneth S. Kosik, Diego Acosta-Alvear, Maxwell Z. Wilson, Lynn Fitzgibbons, Carolina Arias

**Affiliations:** 1 Department of Molecular, Cellular, and Developmental Biology, University of California, Santa Barbara; 2 Neuroscience Research Institute, University of California, Santa Barbara; 3 Student Health Service, University of California, Santa Barbara; 4 Department of Pathology, Santa Barbara Cottage Hospital, Santa Barbara, California; 5 Pacific Diagnostic Laboratories, Santa Barbara, California; 6 Department of Medical Education, Division of Infectious Diseases, Santa Barbara Cottage Hospital, Santa Barbara, California; 7 Center for BioEngineering, University of California, Santa Barbara; 8 Center for Stem Cell Biology and Engineering, University of California, Santa Barbara

## Abstract

**Importance:**

The reopening of colleges and universities in the US during the coronavirus disease 2019 (COVID-19) pandemic is a significant public health challenge. The development of accessible and practical approaches for severe acute respiratory syndrome coronavirus 2 (SARS-CoV-2) detection in the college population is paramount for deploying recurrent surveillance testing as an essential strategy for virus detection, containment, and mitigation.

**Objective:**

To determine the prevalence of SARS-CoV-2 in asymptomatic participants in a university community by using CREST (Cas13-based, rugged, equitable, scalable testing), a CRISPR-based test developed for accessible and large-scale viral screening.

**Design, Setting, and Participants:**

For this cohort study, a total of 1808 asymptomatic participants were screened for SARS-CoV-2 using a CRISPR-based assay and a point-of-reference reverse transcriptase–quantitative polymerase chain reaction (RT-qPCR) test. Viral prevalence in self-collected oropharyngeal swab samples collected from May 28 to June 11, 2020, and from June 23 to July 2, 2020, was evaluated.

**Exposures:**

Testing for SARS-CoV-2.

**Main Outcomes and Measures:**

SARS-CoV-2 status, viral load, and demographic information of the study participants were collected.

**Results:**

Among the 1808 participants (mean [SD] age, 27.3 [11.0] years; 955 [52.8%] female), 732 underwent testing from May to early June (mean [SD] age, 28.4 [11.7] years; 392 [53.6%] female). All test results in this cohort were negative. In contrast, 1076 participants underwent testing from late June to early July (mean [SD] age, 26.6 [10.5] years; 563 [52.3%] female), with 9 positive results by RT-qPCR. Eight of these positive samples were detected by the CRISPR-based assay and confirmed by Clinical Laboratory Improvement Amendments–certified diagnostic testing. The mean (SD) age of the positive cases was 21.7 (3.3) years; all 8 individuals self-identified as students. These metrics showed that a CRISPR-based assay was effective at capturing positive SARS-CoV-2 cases in this student population. Notably, the viral loads detected in these asymptomatic cases resemble those seen in clinical samples, highlighting the potential of covert viral transmission. The shift in viral prevalence coincided with the relaxation of stay-at-home measures.

**Conclusions and Relevance:**

These findings reveal a shift in SARS-CoV-2 prevalence in a young and asymptomatic population and uncover the leading edge of a local outbreak that coincided with rising case counts in the surrounding county and the state of California. The concordance between CRISPR-based and RT-qPCR testing suggests that CRISPR-based assays are reliable and offer alternative options for surveillance testing and detection of SARS-CoV-2 outbreaks, as is required to resume operations in higher-education institutions in the US and abroad.

## Key Points

**Question:**

Are CRISPR-based methods a reliable and accessible option to capture severe acute respiratory syndrome coronavirus 2 (SARS-CoV-2) outbreaks in a college population?

**Findings:**

In this cohort study, 1808 asymptomatic college students were screened for SARS-CoV-2 status using reverse transcriptase–quantitative polymerase chain reaction (RT-qPCR) and CRISPR-based assays. Nine samples positive for SARS-CoV-2 were detected by RT-qPCR, and 8 were confirmed by CRISPR-based assay and clinical laboratory diagnostic testing, uncovering a change in viral prevalence that coincided with the relaxation of lockdown measures and the rise of coronavirus disease 2019 cases in the community.

**Meaning:**

CRISPR-based methods appear to offer reliable SARS-CoV-2 testing for virus screening and allow capture of the leading edge of an outbreak.

## Teaser

This cohort study assesses the prevalence of severe acute respiratory syndrome coronavirus 2 among asymptomatic college students using a new CRISPR-Cas13–based assay.

## Introduction

The coronavirus disease 2019 (COVID-19) pandemic has claimed hundreds of thousands of lives and has disrupted life in countless communities. To control this pandemic, communities worldwide closed businesses, prohibited large social gatherings, and adopted nonpharmacological intervention measures.^1,2,3^ Initial restrictions were successful in several countries where COVID-19 cases, hospitalizations, and deaths declined.^2,3^ However, as communities relaxed social distancing and restrictions, COVID-19 cases returned, often with exponential growth. Several metrics, including percentage of positive test results, hospitalizations, and death rates, have been used to gain insights into epidemic trends in specific populations. Prevalence among asymptomatic persons is an important but more elusive metric, primarily because of test scarcity and prioritization of symptomatic patients or contacts with confirmed cases. Nevertheless, understanding both asymptomatic prevalence and the effect of nonpharmacological intervention measures on infection rates has tremendous potential to inform vital public health decisions.

A fundamental aspect of pandemic control is careful planning for the reopening of college campuses. Although COVID-19 testing has focused on individuals with increased risk of infection and mortality, an increasing disease burden has emerged in those aged 19 to 30 years, many of whom attend colleges and universities.^4^ Every year since 2017, more than 15 million students attend colleges in the US.^5^ Many students reside in dormitories and off-campus housing, frequently in crowded conditions, sharing restrooms, kitchens, and common areas.^6^ These living conditions are associated with high morbidities of diseases such as meningococcal meningitis, influenza, mumps, and measles.^7,8,9,10^ Respiratory pathogens, such as severe acute respiratory syndrome coronavirus 2 (SARS-CoV-2), are easily transmitted among individuals living in college dormitories and during social contact by exposure to live virus in aerosol droplets.^11,12,13^

Further complicating SARS-CoV-2 transmission in university settings is the well-documented infectivity of asymptomatic persons, many of whom are likely to be presymptomatic with high viral loads.^14,15,16,17,18,19,20^ Those without symptoms are likely to be responsible for as many as 44% of new infections.^21^ Recent examples of colleges reopening and promptly closing or implementing drastic quarantine measures for their students after the detection of COVID-19 outbreaks illustrate the challenges of safely bringing academic activities back to campus during a pandemic. The upsurge of cases within college populations also presents a risk beyond campus walls because infections can spill over to neighboring communities.^22^ The early identification of infected individuals through expanded and frequent surveillance testing is essential to curb disease spread. However, before undertaking such large-scale surveillance testing, the prevalence of asymptomatic infection must be ascertained to inform decisions regarding the utility of expanded testing in a university population.^23^

Several methods are currently available for COVID-19 diagnosis, with reverse transcriptase–quantitative polymerase chain reaction (RT-qPCR) assays being most commonly used.^24^ The high demand for COVID-19 testing has overwhelmed supply chains, limiting the availability of critical reagents and specialized equipment necessary for RT-qPCR. CRISPR-based assays provide a robust and sensitive alternative for the detection of SARS-CoV-2 genomes. These assays use common and widely available reagents and are adaptable to minimal instrumentation and infrastructure. Although CRISPR-based tests have been validated for the detection of COVID-19 in clinical samples, no information is available about the performance of these assays for SARS-CoV-2 screening in asymptomatic individuals.^25,26,27,28^

To understand viral prevalence in the university community and to assess the potential of a CRISPR-based test to screen for SARS-CoV-2 in asymptomatic persons, we enrolled healthy volunteers from the University of California, Santa Barbara (UCSB) in a virus screening study. We obtained self-collected oropharyngeal swab samples, processed for SARS-CoV-2 testing using 2 methods: CREST (Cas13-based, rugged, equitable, scalable testing), a newly developed CRISPR-based assay,^25^ and the Centers for Disease Control and Prevention (CDC)–recommended RT-qPCR assay,^29^ which we used as a point-of-reference test. We compared the results obtained from May 28 to June 11, 2020, approximately 2 months into a statewide stay-at-home mandate, and June 23 to July 2, 2020, approximately 3 weeks after easing local restrictions for isolation in the community. Our results revealed no COVID-19 cases in the study population during the May-June collection period. Using the same methods, we demonstrated a substantial shift in prevalence approximately 1 month later, which coincided with changes in community restrictions and public interactions. Notably, the CRISPR-based assay performed as well as the CDC-recommended RT-qPCR assay. Our study substantiates the utility of self-collected oropharyngeal swabs and CRISPR-based testing as valuable alternatives for large-scale surveillance sampling of SARS-CoV-2 in asymptomatic individuals.

## Methods

### Study Population

The population of UCSB includes 26 134 students (82.2%) and 5668 staff and faculty (17.8%). Among the students, 38.2% live in university housing, and 33.6% in the nearby community of Isla Vista (23 096 residents; 1.866 square miles, 12 377 people/square mile). This cohort study was open to all symptom-free individuals 18 years or older who were affiliated with UCSB (students, faculty, staff, and direct relatives). Individuals who exhibited a fever (38.0 °C), cough, or shortness of breath in the 2 weeks before or on the day of sample collection were excluded from the study. Only 5 participants were excluded owing to presenting symptoms at the time of collection and were referred to local health care resources. Preanalytical and postanalytical protocols were reviewed and approved by the institutional review board of Santa Barbara Cottage Hospital. All participants provided written informed consent. This study followed the Strengthening the Reporting of Observational Studies in Epidemiology (STROBE) reporting guideline for cohort studies.

### Sample Collection

Health care professionals at UCSB collected written, informed consent and demographic data (age, address, telephone, sex, and UCSB affiliation) at the sampling locale. Samples were assigned a numeric code for deidentification purposes. Samples were acquired as self-collected oropharyngeal swabs stored in phosphate-buffered saline, with surveillance by a health care professional (H.S., B.M., and L.P.). Samples were inactivated at 56 °C for 30 minutes, and RNA was extracted using 1 of 2 kits (QIAamp MinElute Virus Spin Kit [Qiagen ] or Viral RNA Mini Kit [Qiagen]) from 140 to 200 μL of the sample and eluted in 50 μL.

### SARS-CoV-2 Detection by RT-qPCR 

We performed RT-qPCR following the procedures in the emergency use authorization granted by the US Food and Drug Administration.^29^ Viral RNA was reverse transcribed and amplified using the 1-step complementary DNA master mix kit (TaqPath; Thermo Fisher Scientific 501148245) following the manufacturer’s recommendations. Reactions were prepared as previously described.^25^ Briefly, a 15-μL master mix reaction was prepared using the established CDC protocol,^29^ and 5 μL of RNA were added into the reaction with each of the target-specific RT-qPCR primers and probes. For no-template controls, 5 μL of nuclease-free water were used. Positive control reactions used 10^6^ copies of in vitro transcribed RNA encoding the SARS-CoV-2 nucleocapsid sites N1 and N2. Reactions were run in a qPCR instrument (CFX96 Touch; Bio-Rad Laboratories, Inc) using the following thermal profile: 25 °C for 2 minutes; 50 °C for 15 minutes; 45 cycles of 95 °C for 5 seconds followed by 55 °C for 30 seconds and plate read; and hold at 4 °C. Data were analyzed using the manufacturer’s software (CFX Maestro; Bio-Rad Laboratories, Inc) with a single threshold for determination of quantification cycle (Cq) value. We prepared standard curves of in vitro transcribed RNAs, ranging from 10^6^ to 10^0^ copies/μL, to determine detection limits. One-way analysis of variance with a post hoc Dunnett test was used to determine the Cq value significance from no-template controls using Prism software, version 8 (GraphPad Software Inc). The limit of detection for N1 and N2 is 10^2^ copies/μL (Cq, 32.59 and 34.405, respectively); for ribonuclease P (RNaseP), 10^3^ copies/μL (Cq, 34.328). Samples were considered positive if the signal for both N1 and N2 was above the limit of detection. Samples were processed in-house with a turnaround time from 12 to 30 hours from the moment of collection.

### CREST Assay 

CREST reactions were performed as described.^25^ Briefly, 5 μL of RNA were reverse transcribed using 200 U/μL of reverse transcriptase (RevertAid; Thermo Fisher Scientific) in the presence of murine RNase inhibitor (New England Biolabs). Water was used as the negative control. Positive control reactions used 10^6^ copies of in vitro transcribed RNA. The reaction mixtures were heated to 42 °C for 30 minutes, then placed on ice. We used 2 μL of the resulting complementary DNAs as templates for PCR amplification with *Taq* DNA polymerase (New England Biolabs) using the following thermal profile: 98 °C for 2 minutes; 20 cycles of 98 °C for 15 seconds, 60 °C for 15 seconds, and 72 °C for 15 seconds; and final extension at 72 °C for 5 minutes. Cas13a was used for site-specific detection with fluorescent probes. The reaction was performed in Cas13a cleavage buffer (40mM Tris [pH, 7.5], 1mM dithiothreitol) supplemented with 1mM ribonucleoside triphosphates (Thermo Fisher Scientific), 2 U/μL of RNase inhibitor (New England Biolabs), 0.125μM cleavage reporter (Integrated DNA Technologies, Inc), 1.5 U/μL of T7 RNA polymerase (Lucigen Corporation), 6.3 ng/μL of LwaCas13a, 20nM Cas13 crRNA, and 9mM magnesium chloride. Reactions were composed of 4 μL of Cas13a cleavage solution and 1 μL of the RT-qPCR product in a well of a 384-well plate, with samples run in duplicate or quadruplicate wells. Fluorescence was acquired every 5 minutes for 30 minutes at 37 °C in a qPCR instrument (QuantStudio 5; Applied Biosystems). The initial reading taken at time 0 was subtracted from time 30 minutes to get a difference in relative fluorescence units for each well. To determine a threshold for negative and positive results, the difference in relative fluorescence units from negative control wells was multiplied by 5 and used as a cutoff. The threshold of detection for N1 in CREST is at 38 705 and for N2 is at 29 904. Plates were valid if negative control reactions did not increase 3 times during the experiment. Samples were considered positive if the signal for both N1 and N2 was 5 times greater than the background. Samples were processed in-house with a turnaround time from 12 to 30 hours from the moment of collection. CREST has not yet received emergency use authorization from the US Food and Drug Administration.

### Confirmation of Positive Samples

Samples detected as positive were confirmed by diagnostic testing at the Pacific Diagnostic Laboratories. Positive results were reported to the participants and the Santa Barbara County Public Health Department by Santa Barbara Cottage Hospital clinicians. Participants with confirmed positive test results were offered the opportunity to follow up with clinicians at the UCSB Student Health Service.

### Estimation of Viral Load

To estimate the viral load in the asymptomatic or presymptomatic participants confirmed as having positive test results, the genome equivalents per microliter were calculated based on the Cq values for N1 and N2 from the RT-qPCR assay. The calculation used linear regression on a standard curve ranging from 10^0^ to 10^6^ gene copies/μL.

### Primer, Guide RNA, and Cleavage Reporter Sequences

Primers for reverse transcription and PCR amplification included N1 (F: 5′ gaaatTAATACGACTCACTATAgggcgaccccaaaatcagcgaaat, R: 5′ tctggttactgccagttgaatctg), N2 (F: 5 ’gaaatTAATACGACTCACTATAgggcttacaaacattggccgcaaa, R: 5′ gcgcgacattccgaagaa), or RNaseP (F: 5′ gaaatTAATACGACTCACTATAgggagatttggacctgcgagcg’, R: 5′ gtgagcggctgtctccacaa). Guide RNAs for Cas13 detection included N1 (5′ GAUUUAGACUACCCCAAAAACGAAGGGGACUAAAACaggguccacca aacguaaugcggggugc), N2 (5′ GAUUUAGACUACCCCAAAAACGAAGGGGACUAAAACgcugaagcgcu gggggcaaauugugcaa), or RNaseP (5′ GAUUUAGACUACCCCAAAAACGAAGGGGACUAAAACguccgcgcagagccuucaggucagaacc). CREST cleavage reporter was 6-carboxyfluorescein – (U)_14_ –Blackhole quencher (MilliporeSigma).

### Statistical Analysis

Correlations between N1 and N2 and between our CRISPR-based assay and the RT-qPCR assay were calculated using the Pearson correlation coefficient, assuming data are from a bivariate normal distribution, using the R function cor.test() (R Program for Statistical Computing). Percentage of positive rates were fit using a logistic growth model where current *P* = KP [P + (K – P)e^−rt^], with K = 100%, *P* = .03, r fit by minimizing the error found to be r = 0.101, and rt indicating rate of maximum population growth.

## Results

A total of 1808 healthy volunteers were screened for SARS-CoV-2. All participants were asymptomatic for COVID-19 at the time of sample collection. Samples were collected from May 28 to June 11 (cohort 1 [n = 732]) and from June 23 to July 2 (cohort 2 [n = 1076]). Eight hundred fifty-three participants (47.2%) were male and 955 (52.8%) were female (mean [SD] age, 27.3 [11.0] years). One thousand three hundred and six participants in both cohorts (72.2%) self-identified as UCSB students (519 [70.9%] in cohort 1 and 787 [73.1%] in cohort 2). This population represents the UCSB community with 26 134 students (82.2%) and 5668 staff and faculty (17.8%). Most of the participants (1224 [67.7%]) reported the UCSB neighboring communities of Goleta and Isla Vista as their place of residence. The study population’s mean (SD) age was 28.4 (11.7) and 26.6 (10.5) years for cohorts 1 and 2, respectively, with a minimum age of 18 years and a maximum of 75 years (Table).

**Table.** Characteristics of Individuals Tested for SARS-CoV-2

Characteristic	Study cohort^a^	
	May 28 to June 11, 2020 (n = 732)	June 23 to July 2, 2020 (n = 1076)
**Study population**		
Age, mean (SD), y	28.4 (11.7)	26.6 (10.5)
Age, y		
18-30	556 (76.0)	869 (80.8)
31-50	115 (15.7)	135 (12.5)
51-60	37 (5.1)	47 (4.4)
61-75	24 (3.3)	23 (2.1)
Not reported	0	2 (0.2)
UCSB affiliation		
Student	519 (7.9)	787 (73.1)
Faculty or staff	211 (28.8)	288 (26.8)
Other	2 (0.3)	1 (0.1)
Sex		
Female	392 (53.6)	563 (52.3)
Male	331 (45.2)	506 (47.0)
Other	9 (1.2)	7 (0.7)
Place of residence		
Goleta and Isla Vista	431 (58.9)	793 (73.7)
Santa Barbara	170 (23.2)	185 (17.2)
Other	127 (17.3)	98 (9.1)
Not reported	4 (0.6)	0
**Confirmed positive cases**		
Total	0	8 (0.7)^b^
Age, mean (SD), y	NA	21.7 (3.3)
No. of UCSB students	NA	8
No. with COVID-19 symptoms		
None	NA	2
Mild	NA	2
Classic	NA	2
Not reported	NA	2

Abbreviations: COVID-19, coronavirus disease 2019; NA, not applicable; SARS-CoV-2, severe acute respiratory syndrome coronavirus 2; UCSB, University of California, Santa Barbara.

^a^ Unless otherwise indicated, data are expressed as number (percentage) of participants.

^b^ Cases confirmed by diagnostic testing in a Clinical Laboratory Improvement Amendments–certified laboratory.

**Figure 1.**
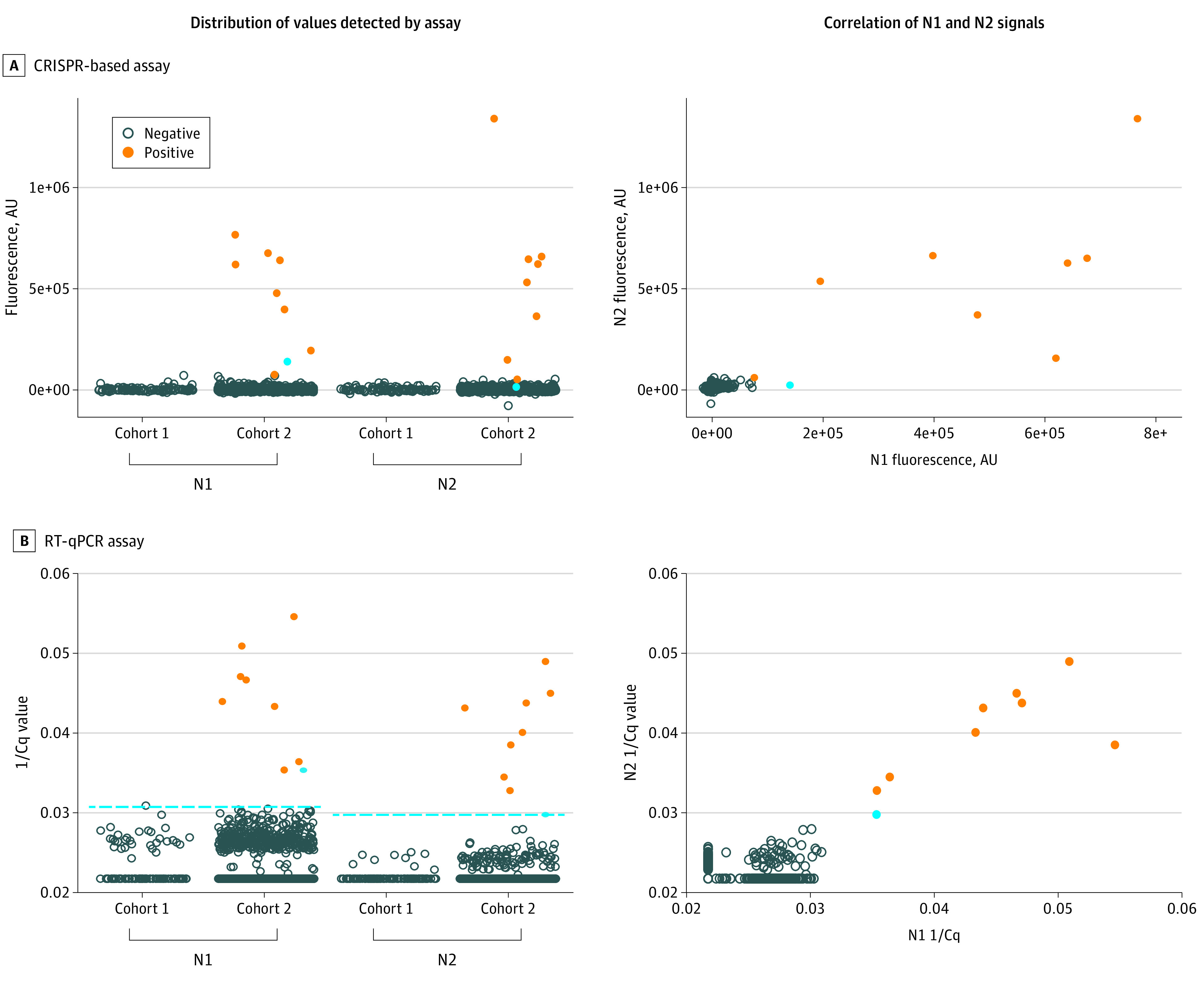
Detection of Positive Samples by CRISPR-Based and Reverse Transcriptase–Quantitative Polymerase Chain Reaction (RT-qPCR) Assays A, Distribution of the fluorescence values by cohort and correlation of nucleocapsid sites N1 and N2 signals by CRISPR-based assay. B, Distribution of the 1/quantification cycle (Cq) values by cohort and correlation of N1 and N2 signals detected by RT-qPCR. Cohort 1 underwent testing from May 28 to June 11, 2020; cohort 2, from June 23 to July 2, 2020. The blue dot indicates 1 sample detected by RT-qPCR but not confirmed by the CRISPR-based assay or by a diagnostic test (note the low level of N2 for this sample). The dashed line indicates the detection limit for RT-qPCR (N1, 1/Cq 0.0306; N2, 1/Cq 0.029). AU indicates arbitrary unit.

**Figure 2.**
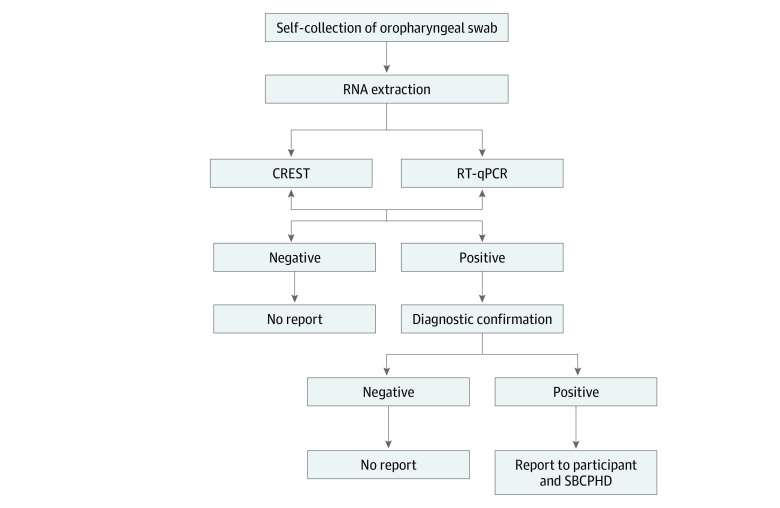
Flow Diagram of Sample Collection and Processing Self-collected oropharyngeal swabs were processed for severe acute respiratory syndrome coronavirus 2 testing using CRISPR-based or reverse transcriptase–quantitative polymerase chain reaction (RT-qPCR) assays. Positive results were confirmed with diagnostic testing in a Clinical Laboratory Improvement Amendments–certified laboratory. Following confirmation, Santa Barbara Cottage Hospital clinicians reported the positive results to the participants and the Santa Barbara County Public Health Department (SBCPHD). CREST indicates Cas13-based, rugged, equitable, scalable testing.

SARS-CoV-2 genomes were detected using CREST, the CRISPR-based method recently developed by Rauch et al,^25^ and the RT-qPCR test recommended by the CDC was used as the point of reference^29^ (eFigure 1 in the Supplement). Both methods detected 2 sites in the nucleocapsid gene, N1 and N2, and 1 site in the host RNaseP transcript, which ensured consistency in the analyses. All samples collected in cohort 1 (n = 732) had negative results by both tests (Figure 1, right side). In contrast, 8 positive samples were detected by the CRISPR-based assay and 9 by RT-qPCR in cohort 2 (n = 1076) (Figure 1). There was a good correlation in detecting the nucleocapsid gene using the N1 and N2 probes (CRISPR-based assay, Pearson correlation coefficient *r* = 0.872) (Figure 1A) and primers (RT-qPCR assay, Pearson correlation coefficient *r* = 0.566) (Figure 1B). The participants with positive results had a mean (SD) age of 21.7 (3.3) years, and all self-identified as UCSB students (Table). The 8 samples detected by the CRISPR-based assay were independently confirmed by a Clinical Laboratory Improvement Amendments–certified laboratory test (Figure 2). One sample had positive results solely by RT-qPCR at the detection threshold, reflecting a low viral copy number (eTable 1 in the Supplement). With this single possible exception, the results obtained by CRISPR-based and RT-qPCR assays were concordant (eFigure 2 in the Supplement).

Six of 8 participants with positive test results for SARS-CoV-2 provided an update of symptoms to the UCSB Student Health Center. Two participants reported no symptoms, 2 reported mild symptoms (nasal congestion, sore throat), and 2 reported classic COVID-19 symptoms (fatigue, anosmia) (Table). None of the participants reported fever as a symptom.

**Figure 3.**
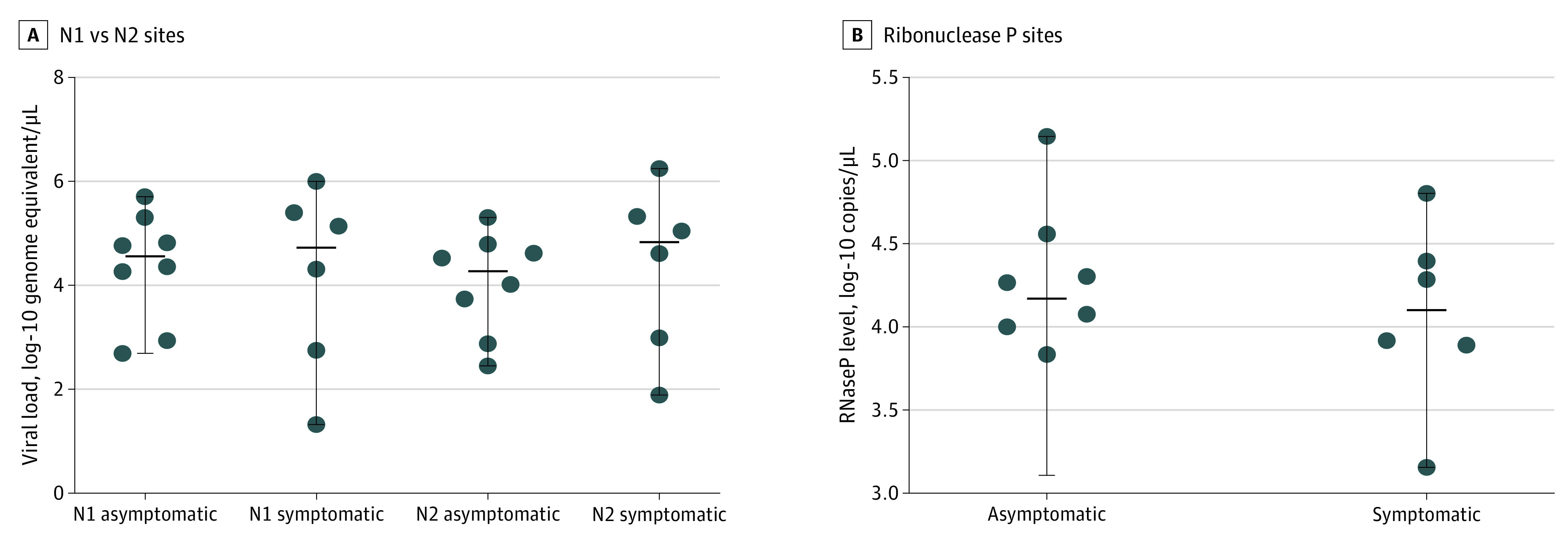
Viral Loads in Asymptomatic and Confirmed Positive Individuals A, Viral loads were calculated using the reverse transcriptase–quantitative polymerase chain reaction (RT-qPCR) data for nucleocapsid sites N1 and N2 detection. B, Ribonuclease P (RNaseP) copies were calculated using the RT-qPCR data for this host gene target. Our analyses included the 8 samples with positive test results we detected in cohort 2, which were confirmed by diagnostic testing. Residual clinical samples from patients with known positive test results (n = 6) provided to us by our collaborators at the Santa Barbara County Public Health Department were used as controls. Solid horizontal lines indicate medians. Differences were not significant between N1 asymptomatic and symptomatic samples (*P* = .95, Mann-Whitney test), N2 asymptomatic and symptomatic samples (*P* = .50, Mann-Whitney test), or asymptomatic and symptomatic RNaseP samples (*P* = .95, Mann-Whitney test).

The estimated viral loads for the positive samples ranged from 286 to 510 000 copies/μL (Figure 3 and eTable 2 in the Supplement). These viral load levels were not significantly different from those detected in a control set of deidentified residual nasopharyngeal swab samples obtained from symptomatic patients in the local community provided to us by collaborators at the Santa Barbara County Public Health Department (eTable 2 in the Supplement and Figure 3). Notably, the quality of the self-collected specimens using oropharyngeal swabs was not significantly different from those collected using nasopharyngeal swabs as measured by the detection of RNaseP transcripts (Figure 3).

**Figure 4.**
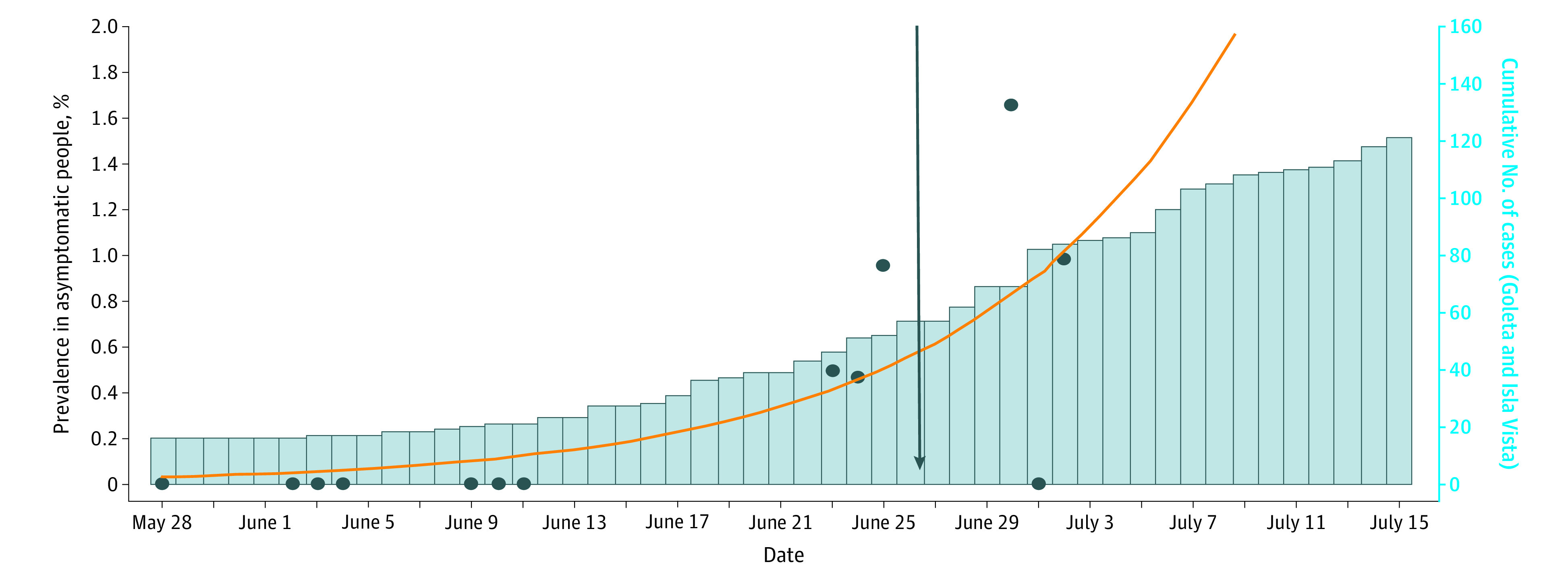
Daily Prevalence of Severe Acute Respiratory Syndrome Coronavirus 2 (SARS-CoV-2) in the Study Population The dots represent daily SARS-CoV-2 prevalence. The trend line, indicated by the orange line, was calculated by finding the *r* in a logistic growth model that minimized the error while fixing the percentage prevalence on May 28 to 0.03%. The blue bars represent the cumulative daily number of diagnosed COVID-19 cases in the Goleta and Isla Vista communities based on official data from the Santa Barbara County Public Health Department. The arrow indicates relaxation of stay-at-home measures in the County of Santa Barbara.

The prevalence of SARS-CoV-2 in the study population in cohort 1 was 0, whereas that of cohort 2 was 0.8%, with a daily incidence ranging from 0 to 1.7% (Figure 4 and eTable 3 in the Supplement). The change in prevalence between cohorts was statistically significant (95% CI, 0.7094-0.7906). The prevalence dynamics in the study population reflect the increase in COVID-19 cases diagnosed in the UCSB neighboring communities of Goleta and Isla Vista, where most of our participants reside (Figure 4). The increase in the number of infections detected in this study—and those in Santa Barbara County—coincided with the reopening of personal care and recreation venues (restaurants and bars) in Santa Barbara County (Figure 4).

## Discussion

As colleges and universities through the US struggle to recover from the academic, social, and economic effects of months of remote learning, a pressing trial remains: how to reopen campuses safely? A primary challenge for university communities is the potential for covert infections promoted by social and academic gatherings, which are unavoidable in the context of a vibrant university campus. Recent evidence indicates that asymptomatic and presymptomatic individuals can unknowingly transmit the virus and fuel covert outbreaks.^19,30,31^ The early detection of asymptomatic infections—particularly those with high SARS-CoV-2 loads, such as those detected in our analyses that may underlie superspreader events—is vital for mitigating viral transmission and containing outbreaks. This information is also essential to guide university directives to make decisions regarding campus openings across the country and ensure superior education continuity. Epidemiological models support this notion and suggest that universal and frequent SARS-CoV-2 testing is necessary for efficient disease containment.^23^ However, the economic effects of providing reliable and regular testing for thousands of students, faculty, and staff may prohibit larger campuses from closely monitoring their communities.

With these considerations in mind, we evaluated the performance of the recently developed CRISPR-based strategy for large-scale viral surveillance in asymptomatic participants. This method, known as CREST, uses PCR amplification and Cas13 for the detection of viral genomes with a simple binary outcome.^25^ This CRISPR-based assay is as efficient at detecting SARS-CoV-2 infections in asymptomatic participants as the CDC-recommended RT-qPCR, which is considered the criterion standard testing method. It also has the added benefit of enabling an easy-to-interpret and dependable binary readout: fluorescence vs no fluorescence. The CRISPR-based assay showed perfect concordance with positive cases diagnosed in a Clinical Laboratory Improvement Amendments–certified laboratory (Pacific Diagnostics Laboratory), further corroborating its robustness. Because CREST was designed to be a low-cost and accessible method, it offers a much-sought alternative for communities where resources are limited and where access to testing is difficult. This CRISPR-based method is scalable, enabling high-throughput testing, and it uses laboratory-generated or off-the-shelf commercially available reagents, thus eliminating the restriction of limiting supply chains. For these reasons, we surmise that CREST can offer a solution for places where access to professional laboratories is restrictive and instances in which a high volume of repetitive sampling is necessary, including the university setting.

One of our most significant observations is the difference in SARS-CoV-2 prevalence between the 2 cohorts we analyzed. We did not detect any infections in the 732 people tested in late May and early June. However, approximately 1 month later, we demonstrated a shift in prevalence, with 8 confirmed cases among 1076 asymptomatic people surveyed. This significant change in the transmission dynamics coincided with the release of community restrictions and increased public and social interactions during the implementation of stage 3 of the California reopening plan in Santa Barbara County. The increase in prevalence was exclusive to young and asymptomatic individuals (mean [SD] age, 21.7 [3.3] years; range, 19-30 years) who self-identified as UCSB students and who may not otherwise have accessed COVID-19 testing. Individuals in this age group are likely to be socially active, highlighting how easily covert infections could result in flare-ups. Our surveillance program detected the initial wave from a local outbreak and coincided with rising case counts in the Goleta and Isla Vista localities, the Santa Barbara County, and the state of California.

### Limitations

This study has some limitations. The reported analytical sensitivity of oropharyngeal swab samples for SARS-CoV-2 detection is lower than that of nasopharyngeal swab samples, particularly when samples are collected 8 to 15 days after onset of illness.^32,33,34,35^ Despite this limitation, we selected self-collected oropharyngeal swabs as the sampling method for SARS-CoV-2 screening. Our goals were to minimize the effect of this study on the limited availability of nasopharyngeal swabs for clinical purposes and reduce the viral exposure of health care personnel who supervised sample collection. The low number of samples with positive results detected herein limits the interpretation of the data. The results presented reflect the low prevalence of SARS-CoV-2 in Santa Barbara County at the time of this study.

## Conclusions

Overall, this cohort study provides evidence supporting the use of CRISPR-based assays as feasible, rapid, and dependable tools for the surveillance of SARS-CoV-2 in asymptomatic individuals. The concordance between RT-qPCR testing and our strategy of using oropharyngeal swabs and CRISPR-based assay substantiates the feasibility of using simpler, equally robust approaches for high-volume recurrent testing, which is a desirable strategy to facilitate the reopening of colleges and universities. Monitoring the population to detect COVID-19 cases before they lead to outbreaks could constitute the paramount containment and mitigation approach within large campus communities and others facing similar challenges.

## Notes

Supplement. **eFigure 1.** Overview of CREST and RT-qPCR Protocols **eFigure 2.** Correlation Between RT-qPCR and CREST Detection of Positive and Negative Samples **eTable 1.** CREST Fluorescence Signal (AU) and RT-qPCR Cq Values for Each of the Positive Samples Detected in the Study **eTable 2.** Viral Load and Known Positive and Negative Samples From the Community **eTable 3.** SARS-CoV-2 Prevalence (Percent of Cases per Day) for Each Collection Day in Cohorts 1 and 2 in the Study
