# Supplementary material for: Comparison of Severe Acute Respiratory Syndrome Coronavirus 2 Screening Using Reverse Transcriptase–Quantitative Polymerase Chain Reaction or CRISPR-Based Assays in Asymptomatic College Students
Source: JAMA Netw Open. 2021 Feb 11;4(2):e2037129. doi: 10.1001/jamanetworkopen.2020.37129 (PMC7879237; doi:10.1001/jamanetworkopen.2020.37129)
Supplement: Supplement. — eFigure 1. Overview of CREST and RT-qPCR Protocols eFigure 2. Correlation Between RT-qPCR and CREST Detection of Positive and Negative Samples eTable 1. CREST Fluorescence Signal (AU) and RT-qPCR Cq Values for Each of the Positive Samples Detected in the Study eTable 2. Viral Load and Known Positive and Negative Samples From the Community eTable 3. SARS-CoV-2 Prevalence (Percent of Cases per Day) for Each Collection Day in Cohorts 1 and 2 in the Study [file jamanetwopen-e2037129-s001.pdf]

Supplementary Online Content

Rauch JN, Valois E, Ponce-Rojas JC, et al. Comparison of severe acute respiratory syndrome coronavirus 2 screening using reverse transcriptase–quantitative polymerase chain reaction or CRISPR-based assays in asymptomatic college students. *JAMA Netw Open*. 2021;4(2):e2037129. doi:10.1001/jamanetworkopen.2020.37129

eFigure 1. Overview of CREST and RT-qPCR Protocols

eFigure 2. Correlation Between RT-qPCR and CREST Detection of Positive and Negative Samples

eTable 1. CREST Fluorescence Signal (AU) and RT-qPCR Cq Values for Each of the Positive Samples Detected in the Study

eTable 2. Viral Load and Known Positive and Negative Samples From the Community

eTable 3. SARS-CoV-2 Prevalence (Percent of Cases per Day) for Each Collection Day in Cohorts 1 and 2 in the Study

This supplementary material has been provided by the authors to give readers additional information about their work.

©2021 Rauch JN et al. *JAMA Network Open*.

eFigure 1. Overview of CREST and RT-qPCR Protocols

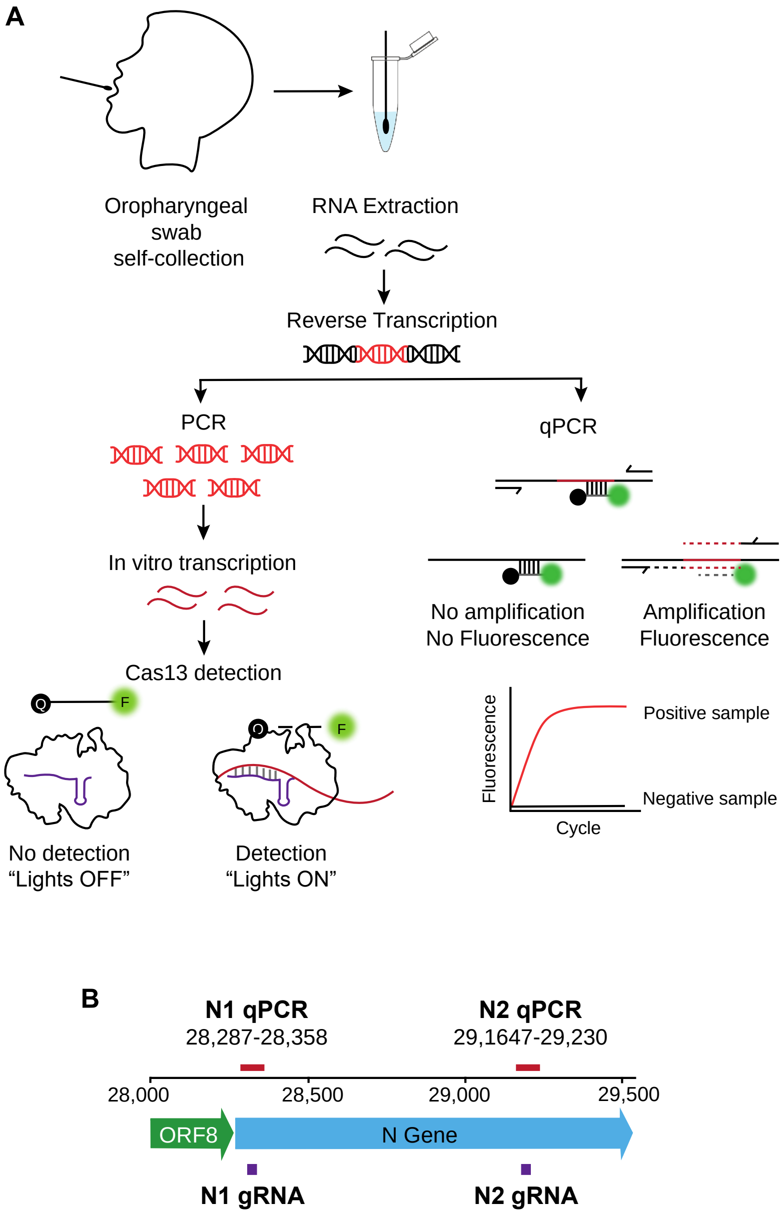

(A) Experimental strategy. Participants collected OP swabs, supervised by a healthcare provider. For CREST, the RNA was reverse transcribed, and the resulting DNA was amplified by the polymerase chain reaction (PCR) using primers for the N1, N2, and RNAse P target regions (see panel B). The PCR-amplified region of interest was

©2021 Rauch JN et al. *JAMA Network Open*.

subsequently transcribed *in vitro* and used as the template for detection by Cas13. The activation of Cas13 following target recognition by the guide RNA (gRNA) was measured using a fluorescent poly-U cleavage reporter. For qPCR, the RNA was reverse transcribed and detected by real-time amplification. (B) Genomic map of the SARS-CoV-2 genome regions detected in this study.

©2021 Rauch JN et al. *JAMA Network Open*.

eFigure 2. Correlation Between RT-qPCR and CREST Detection of Positive and Negative Samples

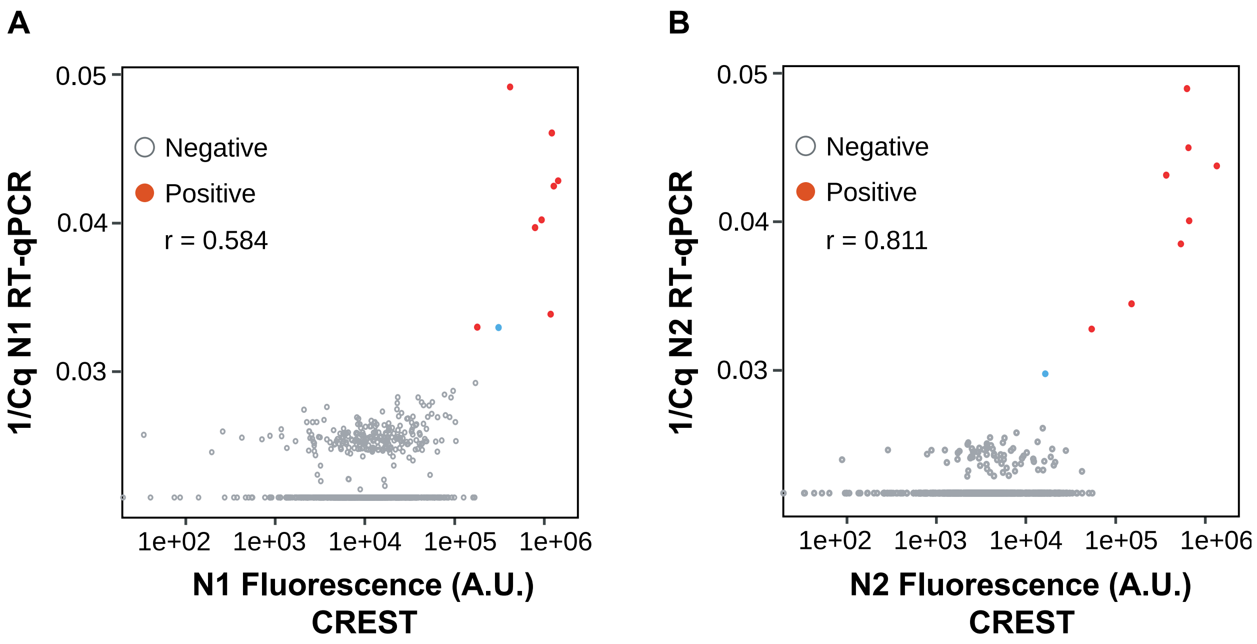

Correlation of the signal detected for N1 (A) or N2 (B) in RT-qPCR and CREST. Grey open dots indicate negative samples; solid red dots indicate positive samples. The blue dot indicates one sample detected by RT-qPCR, but not confirmed by CREST or in a CLIA laboratory test. Pearson correlation coefficient N1 r = 0.584, N2 r = 0.811.

©2021 Rauch JN et al. *JAMA Network Open*.

eTable 1. CREST Fluorescence Signal (AU) and RT-qPCR Cq Values for Each of the Positive Samples Detected in the Study

		CREST (Fluorescence)			RT-qPCR (Cq)		
**Sample**	**Date of collection**	**N1**	**N2**	**RNAseP**	**N1**	**N2**	**RNAse P**
1	6/23/20	767040	1339679	678959	21	23	28
2	6/24/20	620196	150156	730279	27	29	28
3	6/25/20	676364	646462	629386	21	22	23
*4	6/25/20	141479	16355	613756	28	34	26
5	6/25/20	641187	622513	458237	20	20	28
6	6/25/20	398685	659525	345808	23	25	31
7	6/30/20	196032	532165	470316	18	26	29
8	6/30/20	479062	365208	710805	23	23	27
9	7/2/20	77259	53825	450471	28	31	25
Neg. Cont.	N/A	7741	5980	5980	43	43	43
Pos. Cont.	N/A	243665	441170	371064	11	11	18

The negative control is the average signal of all no template samples. The positive control is the average signal for all samples where *in vitro* transcribed RNA was used as a template. The N2 signal for sample 4 was below the limit of detection of CREST, and close to the limit of detection of RT-qPCR. This sample was not confirmed as positive by a diagnostic test performed in a CLIA-certified laboratory.

©2021 Rauch JN et al. *JAMA Network Open*.

eTable 2. Viral Load and Known Positive and Negative Samples From the Community

		Viral load	
**Sample**	**Date of collection**	**N1 (gen. equ./µL)**	**N2 (gen. equ./µL)**
1	6/23/20	67000	42000
2	6/24/20	871	760
3	6/25/20	58500	62700
*4	6/25/20	488	38
5	6/25/20	204000	204000
6	6/25/20	18600	10600
7	6/30/20	510000	5500
8	6/30/20	23000	34000
9	7/2/20	500	286
Pos. Cont. 1	N/A	1006971	1781555
Pos. Cont. 2	N/A	139311	112919
Pos. Cont. 3	N/A	254744	214151
Pos. Cont. 4	N/A	571	989
Pos. Cont. 5	N/A	20866	41588
Pos. Cont. 6	N/A	21	79
Neg. Cont. 1	N/A	0.107	0.001
Neg. Cont. 2	N/A	0.024	0.068
Neg. Cont. 3	N/A	0.127	0.368
Neg. Cont. 4	N/A	0.001	0.302
Neg. Cont. 5	N/A	0.093	0.084
Neg. Cont. 6	N/A	0.012	0.001
Neg. Cont. 7	N/A	0.142	0.001

Viral load (genome equivalents/µL), calculated for the positive samples in this study, and the known positive and negative samples from the community of Santa Barbara County. Sample 4 was not confirmed by diagnostic testing in a CLIA-certified laboratory.

©2021 Rauch JN et al. *JAMA Network Open*.

eTable 3. SARS-CoV-2 Prevalence (Percent of Cases per Day) for Each Collection Day in Cohorts 1 and 2 in the Study

Collection day	Total Samples	Positive Cases	% Prevalence
28-May	41	0	0.000
2-Jun	98	0	0.000
3-Jun	86	0	0.000
4-Jun	108	0	0.000
9-Jun	123	0	0.000
10-Jun	134	0	0.000
11-Jun	141	0	0.000
23-Jun	203	1	0.493
24-Jun	216	1	0.463
25-Jun	314	3	0.955
30-Jun	121	2	1.653
1-Jul	115	0	0.000
2-Jul	102	1	0.980

©2021 Rauch JN et al. *JAMA Network Open*.
